# Subjective cognition trajectories, Alzheimer biomarkers, and incident mild cognitive impairment

**DOI:** 10.1016/j.tjpad.2026.100609

**Published:** 2026-05-29

**Authors:** Elizabeth Kuhn, Luca Kleineidam, Melina Stark, Oliver Peters, Julian Hellmann-Regen, Lukas Preis, Daria Gref, Josef Priller, Eike Jakob Spruth, Maria Gemenetzi, Anja Schneider, Klaus Fliessbach, Jens Wiltfang, Claudia Bartels, Niels Hansen, Ayda Rostamzadeh, Emrah Düzel, Wenzel Glanz, Enise Incesoy, Katharina Buerger, Daniel Janowitz, Sophia Stöcklein, Robert Perneczky, Boris-Stephan Rauchmann, Stefan J. Teipel, Ingo Kilimann, Christoph Laske, Sebastian Sodenkamp, Annika Spottke, Marie Kronmüller, Sandra Roeske, Frederic Brosseron, Alfredo Ramirez, Matthis Synofzik, Matthias C. Schmid, Frank Jessen, Michael Wagner, DELCODE study group

**Affiliations:** a German Center for Neurodegenerative Diseases (DZNE) Bonn, Venusberg-Campus 1/99 53127, Bonn, Germany; b Department of Cognitive Disorders and Old Age Psychiatry, University Hospital Bonn, Venusberg-Campus 1 53127, Bonn, Germany; c German Center for Neurodegenerative Diseases (DZNE) Berlin, Berlin, Germany; d Charité Universitätsmedizin Berlin, Department of Psychiatry and Neurosciences, Hindenburgdamm 30 12203 Berlin, Germany; e Charité Universitätsmedizin Berlin, ECRC Experimental and Clinical Research Center, Lindenberger Weg 80 13125 Berlin, Germany; f Department of Psychiatry and Psychotherapy, Charité, Charitéplatz 1 10117 Berlin, Germany; g Department of Psychiatry and Psychotherapy, School of Medicine and Health, Technical University of Munich, and German Center for Mental Health (DZPG), Munich, Germany; h University of Edinburgh and UK DRI, Edinburgh, United Kingdom; i Department of Psychiatry and Psychotherapy, University Medical Center Goettingen, University of Goettingen, Von-Siebold-Str. 5 37075 Goettingen, Germany; j German Center for Neurodegenerative Diseases (DZNE) Goettingen, Goettingen, Germany; k Neurosciences and Signaling Group, Institute of Biomedicine (iBiMED), Department of Medical Sciences, University of Aveiro, Aveiro, Portugal; l Department of Psychiatry, University of Cologne, Medical Faculty, Kerpener Strasse 62 50924, Cologne, Germany; m German Center for Neurodegenerative Diseases (DZNE) Magdeburg, Magdeburg, Germany; n Institute of Cognitive Neurology and Dementia Research (IKND), Otto-von-Guericke University, Magdeburg, Germany; o German Center for Neurodegenerative Diseases (DZNE) Munich, Feodor-Lynen-Strasse 17,81377 Munich, Germany; p Institute for Stroke and Dementia Research (ISD), University Hospital, LMU Munich, Feodor-Lynen-Strasse 17 81377 Munich, Germany; q Department of Radiology, Ludwig Maximilian University Hospital, Munich 81377, Germany; r Department of Psychiatry and Psychotherapy, University Hospital, LMU Munich, Munich, Germany; s Munich Cluster for Systems Neurology (SyNergy) Munich, Munich, Germany; t Ageing Epidemiology Research Unit (AGE), School of Public Health, Imperial College London, London, United Kingdom; u Department of Neuroradiology, University Hospital, LMU Munich, Munich, Germany; v Sheffield Institute for Translational Neuroscience (SITraN), University of Sheffield, Sheffield, United Kingdom; w German Center for Neurodegenerative Diseases (DZNE) Rostock, Rostock, Germany; x Department of Psychosomatic Medicine, Rostock University Medical Center, Gehlsheimer Str. 20 18147 Rostock, Germany; y German Center for Neurodegenerative Diseases (DZNE) Tuebingen, Tuebingen, Germany; z Section for Dementia Research, Hertie Institute for Clinical Brain Research and Department of Psychiatry and Psychotherapy, University of Tuebingen, Tuebingen, Germany; aa Department of Psychiatry and Psychotherapy, University of Tuebingen, Tuebingen, Germany; ab Clinic for Parkinson's, Sleep and Movement Disorders, Centre for Neurology, University Hospital Bonn, Bonn, Germany; ac Excellence Cluster on Cellular Stress Responses in Aging-Associated Diseases (CECAD), University of Cologne, Joseph-Stelzmann-Strasse 26 50931, Köln, Germany; ad Division of Neurogenetics and Molecular Psychiatry, Department of Psychiatry and Psychotherapy, Faculty of Medicine and University Hospital Cologne, University of Cologne, Cologne, Germany; ae Department of Psychiatry & Glenn Biggs Institute for Alzheimer’s and Neurodegenerative Diseases, San Antonio, TX, USA; af German Center for Neurodegenerative Diseases (DZNE), Tübingen, Germany; ag Division of Translational Genomics of Neurodegenerative Diseases, Hertie Institute for Clinical Brain Research and Center of Neurology, University of Tübingen, Tübingen, Germany; ah Institute for Medical Biometry, Informatics and Epidemiology, University Hospital Bonn, Bonn, Germany

**Keywords:** Subjective cognitive decline, Study partner report, Self-report, Alzheimer’s pathology, Clinical progression

## Abstract

**Background:**

Subjective cognitive decline is common in older adults and may represent an early clinical signal along the Alzheimer’s disease continuum. The clinical relevance of longitudinal changes in subjective cognitive decline remains unclear.

**Objectives:**

To determine whether trajectories of self- or study partner-reported cognitive decline predict progression to mild cognitive impairment and reflect Alzheimer’s disease-specific biological patterns.

**Design, setting, participants:**

Data were pooled from two observational cohorts. Cognitively unimpaired participants with baseline amyloid status, repeated assessments of subjective cognitive decline, and clinical follow-up were included. The study included 770 participants with a median follow-up of 5.0 years (interquartile range 4.0–7.0).

**Measurements:**

Subjective cognitive decline was assessed using the Everyday Cognition questionnaire completed by participants and study partners. Linear mixed-effects models examined associations with amyloid status and progression to mild cognitive impairment. Cox proportional hazards models tested whether one-year changes predicted progression.

**Results:**

Amyloid-positive participants and those who progressed to mild cognitive impairment showed steeper increases in self- and study partner-reported cognitive difficulties over time. Among amyloid-positive participants, only increases in study partner-report differentiated progressors from non-progressors. One-year increases in study partner-report predicted a higher risk of mild cognitive impairment compared with unchanged scores (hazard ratio 3.24; 95% confidence interval 1.73–6.07]), with effects confined to amyloid-positive participants.

**Conclusions:**

Short-term increases in study partner-reported cognitive difficulties identify amyloid-positive cognitively unimpaired older adults at increased risk of near-term progression to mild cognitive impairment. Longitudinal monitoring using study partner reports may provide a low-burden and clinically relevant approach for early risk stratification and surveillance in aging populations.

## 1 Introduction

Subjective cognitive decline (SCD) refers to an individual’s perception of worsening cognition, despite normal performance on standardized cognitive testing [1]. SCD is reported by nearly one-third of adults over 65 [1,2], yet its prognostic significance remains complex. While many individuals remain cognitively stable, a substantial subset experience objective cognitive decline [[3], [4], [5]] and progress to mild cognitive impairment (MCI) or dementia [6,7]. This clinical heterogeneity underscores the need to better distinguish normal aging from early pathological trajectories.

The SCD-Initiative has identified several “SCD-plus” features associated with an increased risk for clinical progression, including the confirmation of decline by a close relative (or study partner [SP]), and, more recently, the persistence of SCD symptoms over time [1,8,9]. Both self- and SP-reports of SCD symptoms independently predict cognitive decline, with combined reports offering greater prognostic value [[10], [11], [12]]. However, as cognitive impairment advances, individuals often lose insight into their cognitive difficulties [13,14], rendering SP-reports increasingly informative in later disease stages.

This evolving dynamic raises a key question: can longitudinal changes in self- and SP-reported SCD symptoms improve early risk prediction in cognitively unimpaired (CU) older adults beyond single-timepoint assessments? Most prior studies have relied on binary, retrospective measures of symptom persistence (e.g., yes/no responses), which may overlook subtle or gradual changes [15,16]. In contrast, quantitatively tracking symptom severity over time could provide a more sensitive and dynamic indicator of risk.

Preliminary findings from the SCIENCe cohort showed increasing self- and SP-reported SCD symptoms in individuals who progressed to MCI or dementia [17], with SP-reported symptoms increasing specifically among those with abnormal amyloid-beta (Aβ) levels, a hallmark biomarker of the Alzheimer’s disease (AD) biological continuum [18,19]. These findings suggest that longitudinal increases in SP-reported SCD symptoms may be especially sensitive to underlying AD pathology [20]. Yet, it remains unclear whether such trajectories specifically characterize individuals who subsequently experience clinical progression, and whether short-term changes provide incremental prognostic value beyond baseline symptom levels.

In this study, we aim to validate and extend these findings across independent cohorts. We examine whether longitudinal trajectories of self- and SP-reported SCD symptoms are associated with progression to MCI in CU individuals, including those with biomarker-confirmed AD pathology (i.e., abnormal Aβ alone or in combination with tau). We further test whether short-interval changes in SCD symptom severity (e.g., over one year), particularly increasing SP-reported symptoms relative to self-reported symptoms, can enhance prediction of clinical progression beyond baseline assessments alone, thereby extending the concept of persistence toward a more dynamic characterization of SCD trajectories. These insights could refine early risk stratification, inform targeted clinical surveillance, and support timely interventions in individuals at greatest risk for neurodegeneration or cognitive decline.

## 2 Methods

### 2.1 Study design

Data were obtained from two longitudinal observational studies: the *German Center for Neurodegenerative Diseases (DZNE) Longitudinal Cognitive Impairment and Dementia Study* (DELCODE, 10 university-based memory centers in Germany) and the *Alzheimer’s Disease Neuroimaging Initiative* (ADNI, 63 sites in North America, http://adni.loni.usc.edu, data retrieval on September 17, 2023 [ADNI2 and ADNI3 phases]). Both studies were approved by local ethics committees and institutional review boards, and participants provided written informed consent. DELCODE is registered with the German Clinical Trials Register (nb. DRKS00007966, 04/05/2015) and ADNI with http://clinicaltrials.gov (nb. NCT00106899). This report adheres to STROBE guidelines for observational cohort studies.

### 2.2 Participants

All selected participants were CU older adults (DELCODE, *N* = 490; ADNI, *N* = 280) enrolled between May 5, 2011, and November 10, 2021, who had (1) no evidence of objective cognitive impairment, (2) baseline mini mental state examination (MMSE) scores of 24–30, (3) functional activities questionnaire scores ≤9, (4) Aβ status available at baseline, and (5) underwent a clinical evaluation and completed self- and SP-reported SCD questionnaires at both baseline and at least one follow-up visit (see details in Sections 2.3–2.5). Median (IQR) follow-up was 5.0 (4.0–7.0) years (DELCODE, 5.1 [4.0–6.6]; ADNI, 4.2 [3.0–7.5]), with a range of 1.0–8.5 years. Most participants were community-recruited CU individuals recruited via public advertisements; a subset of 279 DELCODE participants were patients with SCD recruited from memory clinics after reporting cognitive concerns to the referring physician while remaining CU based on standardized cognitive assessment. Detailed inclusion and exclusion criteria are detailed in **eMethods** and reported elsewhere [[21], [22], [23]].

### 2.3 Subjective cognitive decline assessments

SCD symptom severity was assessed using the Everyday Cognition Questionnaire (Ecog) [24] in both cohorts, a 39-item questionnaire in which participants (self-report) and their close relative (SP-report) rate the participant’s current ability to perform everyday tasks now compared to 10 years ago on a 4-point scale ranging from “no change” to “consistently worse” (“I don’t know” responses were treated as missing). A global mean Ecog scores across all completed items were calculated; therefore, scores ranged from 1 to 4, with higher scores indicating greater SCD symptom severity.

To ensure reliable composites scores, observations with more than 15% missing Ecog items (>5 items) were excluded. For the remaining observations, missing item-level data were imputed to allow calculation of total scores using multilevel multiple imputation (*mice* package*, 2l.pan* method for longitudinal data), with values constrained to the original response range (1–4) to account for the hierarchical structure of repeated measures and preserve within-person variability, rather than relying on mean substitution.

A cognitive awareness index (CAI) was calculated as the difference between self- and SP-reports. Positive CAI scores indicated that the participant provided a higher rating of their difficulties compared to what their SP-reported, whereas negative scores indicated lower ratings, likely reflecting lower awareness [25]. Cumulative SCD reports score were also explored by summing self- and SP-reports [3].

To quantify short-term changes, one-year difference score (hereafter called DC1) was derived by subtracting the baseline from the first follow-up score for each SCD measure. This measure was then weighted to account for the time interval between the two time points (i.e., range considered: 6–18 months). These continuous DC1 scores were categorized into “increased”, “decreased,” or “unchanged” (reference) subgroups based on whether intra-individual change exceeded ± 5% of each measure’s total range (e.g., ± 0.15 for mean Ecog; ± 0.30 for CAI and cumulative scores). Participants were thus considered “unchanged” when score variations remained within a small range likely reflecting minor fluctuations rather than meaningful symptom change. DC1 values were available in a subset of 353 (45.8%) participants (DELCODE, 215 [43.9%]; ADNI, 138 [49.3%]).

### 2.4 Clinical progression to incident-MCI

Clinical progression to incident-MCI was determined through consensus clinical review in DELCODE (biomarker- and genetic-blinded) [26], and physician diagnoses in ADNI. A total of 131 (17.0%) participants progressed to MCI during follow-up (DELCODE, *N* = 96 [19.6%]; ADNI, *N* = 35 [12.5%]). Further details are available in **eMethods**.

### 2.5 Alzheimer’s disease biomarkers

Initial-stage biomarkers of Aβ and tau were assessed using multiple modalities. Among 770 participants, amyloid status was determined by Aβ-PET ([18F]florbetapir [*N* = 221, 28.7%] or [18F]florbetaben [FBB, *N* = 59, 7.7%]), or CSF- (*N* = 274, 35.6%) or plasma-derived Aβ_42/40_ ratio (*N* = 216, 28.1%). Core 1 tau status was also available in 710 participants, based on tau-PET ([18F]flortaucipir [FTP, *N* = 106, 14.9%]), or CSF- (*N* = 424, 59.7%) or plasma-derived ptau_181_ levels (*N* = 180, 25.4%). Aβ+ or T1+ classifications (abnormally elevated Aβ and/or tau levels) followed cohort-specific published thresholds [[27], [28], [29], [30], [31], [32], [33], [34], [35], [36], [37]], except DELCODE plasma ptau_181_ dichotomized by Youden index (CSF ptau_181_ as reference). Overall, 239 (31.0%) participants were Aβ+, and 77 (10.9%) were Aβ+T1+. All procedures adhered to standardized cohort-specific protocols (see **eMethods**) [[27], [28], [29], [30], [31],^33^].

### 2.6 Statistical analyses

Baseline demographic, clinical, and cognitive differences were tested with Kruskall-Wallis and post-hoc Dunn tests for non-normally distributed continuous variables, and χ^2^ tests for categorical variables, across four subgroups: Aβ-Stable, Aβ+Stable, Aβ-iMCI and Aβ+iMCI, with up to eight years of follow-up.

To address our first objective, linear mixed-effects models (*lme4* package in R) [38] with random intercepts and slopes for time (years from baseline) were used to model longitudinal changes in Ecog self- and SP-reports. Fixed effects included clinical progression to incident-MCI (model 1), baseline Aβ status (model 2), and their interaction (model 3). Three-way interactions (time x Aβ x clinical progression) were further examined with Bonferroni-corrected post hoc contrasts to test for slope differences within and across subgroups (*ggeffects* package in R) [39]. Model 1 was also replicated in Aβ stratified subgroups to determine specificity to AD. In these models, clinical progression to iMCI was modeled as a binary grouping variable in longitudinal analyses.

To address the second objective, multivariable Cox proportional hazards regression models (*survival* package) tested whether one-year changes in SCD symptom severity (categorical DC1, Section 2.3) predicted clinical progression to incident-MCI (outcome, time to progression in years from the first follow-up censored at the last available assessment; model 4), and whether associations differed by baseline Aβ status (model 5). Participants who had already converted to MCI at the first follow-up visit were excluded, as one-year SCD changes could not be interpreted as predictors of subsequent clinical progression in these individuals.

To isolate the incremental predictive value of DC1 beyond baseline SCD symptoms severity, multivariate Cox models including both terms were used. Kaplan-Meier curves were used for visualization. Additional multivariable models tested independent effects of self- and SP-reports.

Complementary analyses explored (1) combined SCD reports (CAI- and cumulative-Ecog scores) versus individual reports; and (2) whether SCD trajectories could be specific to Aβ+T_1_+ participants.

To maximize sample size, analyses were performed first in the combined sample and then repeated in stratified cohorts. All models were adjusted for age, sex, years of education, and cohort. Because Aβ and tau definitions and recruitment settings varied, these variables were added as covariates when relevant. Mixed models were additionally adjusted for the interaction of these covariates with time. Analyses were conducted using R 4.2.3 (R Foundation) from August 2022 to December 2024, with Bonferroni correction for multiple comparison across four SCD measures per model (α=0.0125).

## 3 Results

### 3.1 Participant characteristics

Overall, 770 participants were included (median age [IQR]: 69.9 [66.0–74.6] years; 405 [52.6%] female). Participants were categorized as Aβ-stable (*n* = 462, 60.0%), Aβ+stable (*n* = 177, 23.0%), Aβ-iMCI (*n* = 69, 9.0%), and Aβ+iMCI (*n* = 62, 8.1%). Participants characteristics are summarized in Table 1 and **eTables 1–2**.

**Table 1:** Baseline participants demographics in the combined sample (*N* = 770) according to amyloid status and clinical progression to MCI.

	Aβ-Stable	Aβ+Stable	Aβ-iMCI	Aβ+iMCI	Overall	P value
No. (%)	462 (60.0%)	177 (23.0%)	69 (9.0%)	62 (8.0%)	770 (100%)	
FU time, median (IQR), y	5.0 (4.0–7.0)	5.0 (3.7–6.5)	4.4 (3.9–6.1)	4.7 (3.4–6.0)	5.0 (4.0–7.0)	*.07*
Age, median (IQR), y	68.4 (66.8–76.4)	71.5 (69.9–78.5)	71.0 (67.4–76.2)	74.6 (69.9–78.5)	69.9 (66.0–74.6)	<0.001
Female, No. (%)	267 (57.8%)	79 (44.6%)	35 (50.7%)	24 (38.7%)	405 (52.6%)	.002
Education, median (IQR), y	16 (13–18)	16 (13–18)	14 (12–17)	15 (13–17.5)	16 (13–18)	*.09*
APOEe4 carrier*, No. (%)	85 (18.7%)	94 (54.0%)	16 (23.2%)	33 (54.1%)	228 (30.0%)	<0.001
MMSE score, median (IQR)	30 (29–30)	30 (29–30)	29 (28–30)	29 (28.5–30)	30 (29–30)	<0.001
Memory clinic, No. (%)	148 (32.0%)	60 (33.9%)	36 (52.2%)	35 (56.5%)	279 (36.2%)	<0.001
No. (%) with tau status	427 (60.1%)	164 (23.1%)	65 (9.2%)	54 (7.6%)	710 (100%)	
Tau status T+, No. (%)	45 (10.5%)	12 (18.5%)	52 (31.7%)	25 (46.3%)	134 (18.9%)	<0.001

⁎ Only 759 participants (7 missing in Aβ-Stable, 3 missing in Aβ+Stable, and 1 missing in Aβ+iMCI) had available APOE data. Abbreviations: Aβ-, participants amyloid-negative at baseline; Aβ+, participants amyloid-positive at baseline; FU, Follow-up, iMCI, participants that progressed to incident mild cognitive impairment during the follow-up period; IQR, interquartile; MMSE, mini mental state examination; Stable, participants cognitively stable during the follow-up period; T+, participants tau-positive at baseline.

Briefly, compared with Aβ-stable participants, both Aβ+ groups included fewer females and higher proportions of APOE ε4 carriers. Participants who progressed to incident MCI were more frequently recruited from memory clinics and had lower baseline MMSE scores, regardless of Aβ status. Tau positivity was more prevalent in Aβ+iMCI than in other groups, and also in Aβ+stable than in Aβ-stable participants. Education level and follow-up duration did not differ across groups.

### 3.2 Longitudinal changes in Ecog reports

**Fig. 1:**
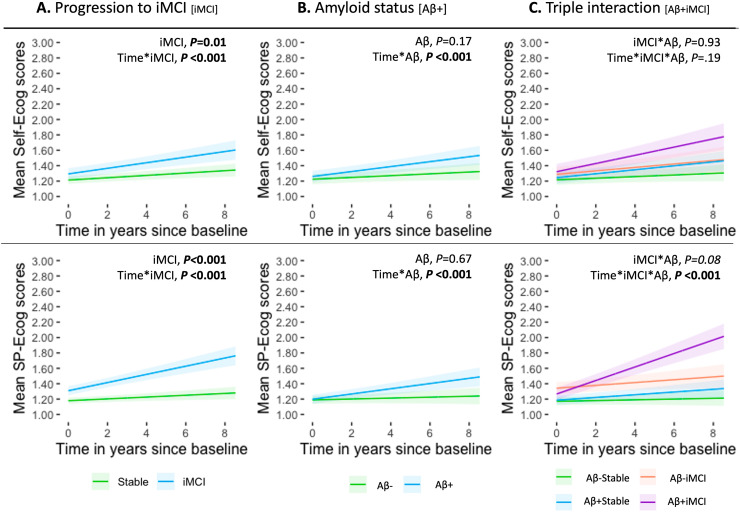
Longitudinal changes in SCD reports according to clinical progression and amyloid status. Predicted changes over time in self- and study partner (SP)-reported subjective cognitive decline (SCD) in cognitively unimpaired (CU) participants are shown according to: (A) clinical progression to mild cognitive impairment (iMCI) during the follow-up period, (B) presence of amyloid pathology (Ab+) at baseline, and (C) their interaction. The estimates illustrated here were generated using the ggeffects package of the R software.

At baseline, the iMCI group showed significantly higher Ecog scores than the Stable group (self-report: est.=0.08, SE=0.03, *P* = 0.01; SP-report: est.=0.13, SE=0.03, *P* < 0.001; Fig. 1**A**). No baseline differences were observed by Aβ status (Self-report: est.=0.04, SE=0.03, *P* = 0.17; SP-report: est.=0.009, SE=0.02, *P* = 0.67; Fig. 1**B**), in independent models.

Over time, both iMCI and Aβ+ groups demonstrated significantly steeper increases in Ecog scores compared to their respective reference groups (iMCI vs. Stable: Self-report est.=0.02, SE=0.006; SP-report est.=0.04, SE=0.006; Aβ+ vs. Aβ-: Self-report est.=0.02, SE=0.005; SP-report est.=0.03, SE=0.005; all *P* < 0.001).

A significant three-way interaction between time, clinical progression, and Aβ status, was observed for SP-report Ecog scores only (est.=0.06, SE=0.01, *P* < 0.001; Fig. 1**C**), with the steepest longitudinal increases observed in the Aβ+iMCI group (**eTable3**). Stratified analyses confirmed a significant time-by-progression interaction within the Aβ+ subgroup (est.=0.07, SE=0.01, *P* < 0.001; **eTable4**). SP-Ecog scores increased over time in all Aβ+ subgroups but remained stable in Aβ- subgroups (**eTable3**). No significant baseline differences in Ecog scores were found among the four groups (Self-report: est.=0.006, SE=0.06, *P* = 0.93; SP-report: est.=−0.09, SE=0.05, *P* = 0.08). Individual trajectories are illustrated in spaghetti plots (**eFigure1)**.

Findings were broadly consistent across cohorts. In DELCODE, all main effects and interactions remained, including within the SCD subsample. In ADNI, effects surviving Bonferroni correction included the interaction between time and Aβ status on self-Ecog (est.=0.03, SE=0.009, *P* = 0.004), and the interaction between time and clinical progression on SP-Ecog scores (baseline: est.=0.17, SE=0.05, *P* < 0.001; slope: est.=0.06, SE=0.02, *P* < 0.001), particularly within the Aβ+ subgroup (est.=0.07, SE=0.03, *P* = 0.012; **eTables 3–5**).

### 3.3 Risk of progression to MCI risk according to one-year Ecog changes

**Fig. 2:**
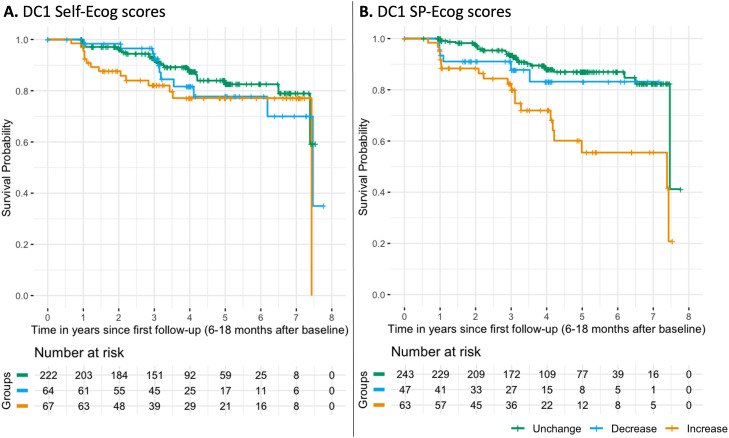
Risk of clinical progression to incident-MCI according to one-year changes in SCD reports. Kaplan-Meier curves illustrating the risk of clinical progression to incident-MCI (survival probability) from the first follow-up according to categorical DC1 scores. Groups corresponds to unchanged, decreased and increased levels of self- and study partner (SP)-Ecog scores over one-year using a 5% change threshold.

DC1 SP-Ecog scores predicted future clinical progression to iMCI (*P* = 0.002), driven by increased scores (N_event_=20; HR [95% CI]=3.24[1.73–6.07]; *P* < 0.001). Decreased scores showed no increased risk compared to unchanged scores (N_event_=6; HR [95% CI]=1.35[0.50–3.61]; *P* = 0.55). Neither DC1 self-Ecog (*P* = 0.13), nor baseline self- (HR [95% CI]=1.29[0.50–3.35]; *P* = 0.59) or SP-Ecog scores (HR [95% CI]=1.11[0.43–2.85]; *P* = 0.83) predicted risk (Fig. 2).

In Aβ-stratified models, increased DC1 SP-Ecog scores predicted progression in Aβ+ participants (N_event_=12; HR [95% CI]=4.18[1.80–9.73]; *P* < 0.001), but not in Aβ- participants (*P* = 0.13). However, the interaction between DC1 SP-Ecog and Aβ status was not significant (*P* = 0.50). Similar trends appeared in stratified cohort analyses, although not all survived Bonferroni correction. Nothing significant was found for DC1 self-Ecog scores (**eTable6**).

In multivariate Cox models including both SP- and self-Ecog, increased DC1 SP-Ecog remained significantly associated with progression (N_event_=20; HR [95% CI]=3.93[1.82–6.49]; *P* < 0.001; **eTable7**).

### 3.4 Complementary analyses

#### 3.4.1 Combined SCD reports

In linear mixed-effects models, both the iMCI and Aβ+ groups showed steeper increases in Cumulative-Ecog scores compared to Stable (est.=0.06, SE=0.009, *P* < 0.001) and Aβ- (est.=0.04, SE=0.007, *P* < 0.001) groups. No Bonferroni-significant effects were found for CAI-Ecog scores.

**Fig. 3:**
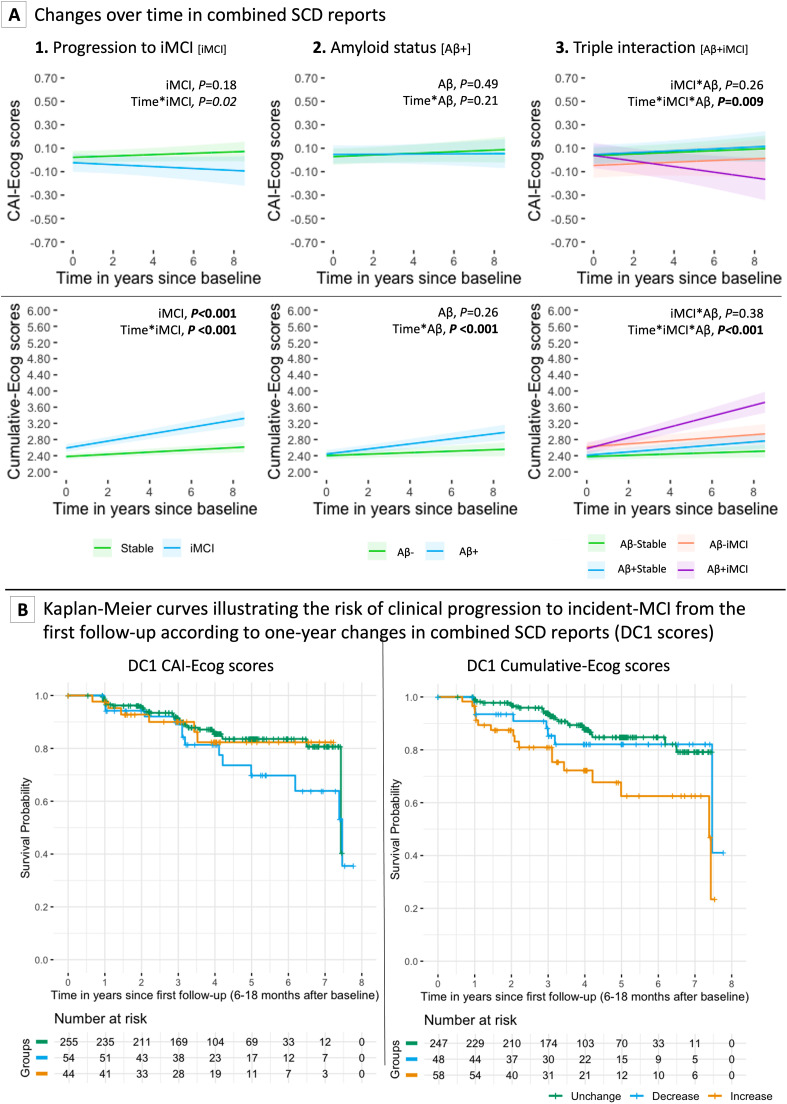
Longitudinal changes in combined SCD reports and risk of clinical progression to MCI. (A) Predicted longitudinal changes in combined subjective cognitive decline (SCD) measures among cognitively unimpaired (CU) individuals, stratified by (1) clinical progression to incident mild cognitive impairment (iMCI), (2) amyloid-β (Aβ) status at baseline, and (3) their interaction. Two SCD dimensions are presented: CAI-Ecog (discrepancy between SP– and self-reported Ecog scores) and cumulative-Ecog (combined SP- and self-reported Ecog scores). Models were adjusted for age, sex, education, and baseline Ecog scores. Shaded areas represent 95% confidence intervals. Estimates were derived using the ggeffectspackage in R. (B) Kaplan–Meier survival curves showing the risk of progression to iMCI based on one-year changes in combined SCD reports. Participants were categorized into "Decrease", "Unchanged", or "Increase" groups based on changes in CAI-Ecog (left panel) and cumulative-Ecog (right panel) scores between baseline and first follow-up (6–18 months). Risk groups were associated with subsequent time to progression to iMCI during longitudinal follow-up.

A significant three-way interaction among time, clinical progression, and Aβ status was observed, indicating a sharper decrease in CAI-Ecog scores (est.=−0.03, SE=0.01, *P* = 0.009) and increase in Cumulative-Ecog scores (est.=0.07, SE=0.02, *P* < 0.001) in the Aβ+iMCI group versus all others, and for Aβ+Stable versus Aβ-Stable (Fig. 3**A; eTable3**). Stratified analyses by Aβ status confirmed that these interactions were driven by Aβ+ participants (**eTable4**).

In Cox regression, neither DC1 Cumulative-Ecog nor CAI-Ecog scores significantly predicted progression to MCI (*P* = 0.02 and *P* = 0.59, respectively; Fig. 3**B; eTable6**).

#### 3.4.2 By initial-stage AD biomarkers

**Fig. 4:**
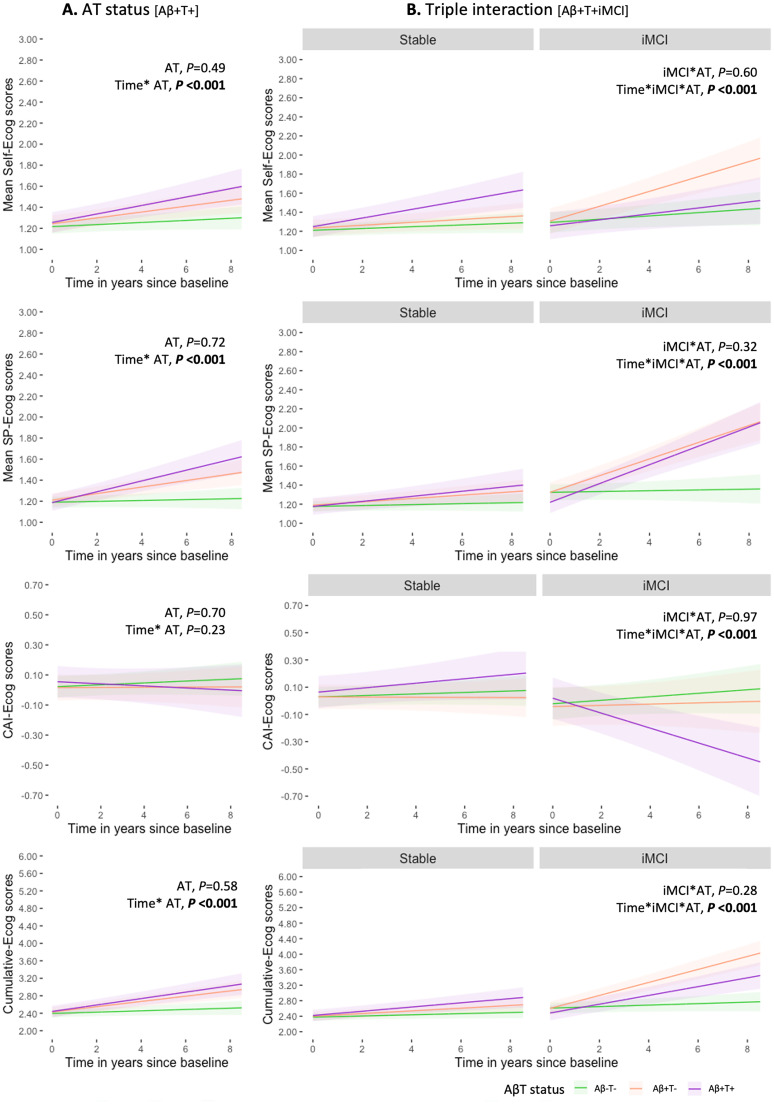
Longitudinal changes in SCD reports by clinical progression and AβT status. Predicted changes over time in self- and study partner (SP)-reported subjective cognitive decline (SCD) among cognitively unimpaired (CU) participants are shown based on baseline amyloid and tau positivity (Ab+T+; A), and its interaction with clinical progression to incident mild cognitive impairment (iMCI) during the follow-up period (B). Estimates and 95% confidence intervals were derived using the ggeffects package in R.

Among participants with available tau data, longitudinal trajectories were examined according to baseline Aβ and tau status, using Aβ-T_1_- as the reference group. Significant interactions between time and AβT status were observed across all Ecog scores (all *P* < 0.001), except CAI-Ecog (*P* = 0.23**;** Fig. 4**A; eTable8**). Both Aβ+T_1_- and Aβ+T_1_+ groups showed steeper longitudinal increases in self- and SP-reports compared with Aβ-T_1_-.

A significant three-way interaction (time x progression x AβT) was observed across all Ecog measures (Fig. 4**B,** all *P* < 0.001). Bonferroni-adjusted post hoc comparisons indicated that both Aβ+T_1_+ and Aβ+T_1_- iMCI groups differed significantly from Stable groups and from Aβ-T_1_- iMCI on SP- and cumulative-Ecog scores (all P_adj_<0.002). For self-Ecog, differences were specific to the Aβ+T_1_- iMCI group, and for CAI-Ecog, to the Aβ+T_1_+ iMCI group (**eTable8**). Due to low event rates, Cox models stratified by AβT status were not performed.

## 4 Discussion

In this longitudinal study of CU older adults, we examined how trajectories of self- and SP-reported cognitive changes relate to baseline AD biomarkers (Aβ and tau) and subsequent progression to MCI. Across up to 8-years of follow-up, SP-reported cognitive difficulties increased more steeply in Aβ+ individuals who later progressed to MCI, whereas self-reported changes did not distinguish Aβ+ progressors from non-progressors. Importantly, similar patterns were observed over shorter time intervals: one-year increases in SP-reports predicted near-term clinical progression, with effects most evident among biomarker-positive individuals who had not yet converted during that one-year period.

At baseline, higher SCD symptom severity (whether reported by participants themselves or by SP-) were observed among individuals who later progressed to MCI, regardless of amyloid status. In contrast, amyloid-positivity alone was not associated with greater SCD symptoms at study entry. Over time, however, both Aβ+ participants and clinical progressors exhibited steeper increases in self- and SP-reported SCD symptoms compared to Aβ- and clinically stable individuals. These findings align with prior studies suggesting that subtle functional-cognitive changes may be noticed by individuals and their close relatives’ years before formal diagnostic thresholds are reached [11,[40], [41], [42]]. Beyond validating this observation, our findings may also help clarify inconsistencies in prior cross-sectional studies of SCD symptoms and Aβ status [[43], [44], [45], [46], [47]]. They suggest a temporal dissociation whereby SCD symptoms severity, particularly as perceived by close relatives, may gradually intensify after amyloid reaches pathological levels and become increasingly informative and detectable as the risk of clinical progression rises. This pattern supports the notion that cross-sectional SCD symptoms severity and longitudinal change may capture distinct stages of the AD continuum [17,48,49]. Further studies combining serial biomarker assessments with dynamic measures of cognition and awareness will be needed to clarify these temporal relationships.

A central finding of this study is that longitudinal SP-reported cognitive changes were more closely associated with clinical progression than self-reported changes in Aβ+ individuals. While both self- and SP-reports reflected increasing perception of cognitive difficulties in the presence of AD pathology, only SP- trajectories consistently differentiated those who later progressed to MCI. Informant reports are routinely used in clinical practice to characterize functional decline; our findings extend this principle by demonstrating that the rate of change in SP-reported difficulties provides prognostic information beyond baseline biomarker status, and does so well before diagnostic criteria for MCI are met. These results reinforce the added value of close relative perspectives in early disease tracking, and extend previous findings linking SP-reports to amyloid burden [20], [43], [50]. Together, they suggest that longitudinal SP-based monitoring may help identify individuals at particularly high short-term risk of cognitive decline within the biologically defined AD continuum.

To further enhance clinical and public-health relevance, we examined short-term changes in perceived cognition. Previous studies have shown that persistence of self-reported SCD symptoms increases the likelihood of subsequent cognitive decline, but these approaches typically rely on binary indicators [15,16]. Extending this work, we show that one-year increases in SP-reported SCD symptoms severity were associated with a three- to four-fold increased risk of progression to MCI among Aβ+ individuals, whereas no such associations were observed in the Aβ- group (although no significant interaction with amyloid status) or with self-report. These findings, consistent with recent evidence from the SCIENCe cohort [17], support short-term SP-based change as a dynamic “SCD-plus” feature and highlight its potential value for early risk stratification in aging populations [1,8,9,51].

We also explored composite metrics integrating self- and SP-reports that have been used in previous studies [3,25]. While the Cumulative-Ecog score largely mirrored SP-report trajectories and added limited predictive value, increasing divergence between self- and SP-report (as captured by the CAI-Ecog) emerged over time among Aβ+T1+ participants who progressed to MCI. This pattern, driven by SP-reports increasingly outweighing self-perceptions, may reflect early loss of insight into cognitive difficulties accompanying AD-related neurodegeneration. However, short-term changes in CAI-Ecog did not predict clinical progression, suggesting that awareness-related measures may be more informative for characterizing disease stage than for near-term risk prediction.

Strengths of this study include the use of two large, well-characterized multicenter cohorts (DELCODE and ADNI), prospective clinical follow-up, and biomarker-confirmed AD risk stratification. The combination of long-term trajectory modeling with short-term change analyses allowed us to capture both gradual and more proximal signals of risk. However, several limitations should be acknowledged. Despite statistical adjustment and stratified analyses, pooling cohorts with differing recruitment procedures and biomarker assessment methods may have introduced residual heterogeneity. In addition, the sample was highly educated and predominantly of European ancestry, and the reliance on SP-based measures may further limit generalizability by excluding individuals without available social support. Short-term analyses were based on smaller subsets with more limited availability of tau data and dementia conversions, while AD biomarkers were only available at baseline, precluding evaluation of how evolving AD pathology relates to SCD trajectories.

In conclusion, longitudinal increases in SP-reported SCD symptom severity, particularly over short intervals, are associated with subsequent progression to MCI among amyloid-positive CU older adults. From a healthy aging perspective, these findings highlight the potential relevance of SP-based longitudinal monitoring as a low-burden approach to identifying individuals at increased short-term risk of cognitive decline before clinically meaningful impairment emerges. While further studies are needed to confirm these findings across diverse populations and settings, incorporating short-term SP-reported changes into early detection frameworks may help support more timely clinical surveillance and targeted preventive strategies aimed at delaying clinically meaningful cognitive decline in aging populations.

## Funding sources

EK was funded by the Fondation Philippe Chatrier and the Helmholtz Artificial Intelligence Cooperation Unit. MS was funded by a Hertie Network of Excellence in Clinical Neuroscience research grant awarded to LK (P1230010).

## Declaration of generative AI and AI-assisted technologies in the manuscript preparation process

During the preparation of this work the authors used ChatGPT (GPT-5, OpenAI, San Francisco, CA, USA) in order to assist in reducing word count and improving language clarity. After using this tool/service, the authors reviewed and edited the content as needed and take full responsibility for the content of the published article.

## CRediT authorship contribution statement

**Elizabeth Kuhn:** Writing – review & editing, Writing – original draft, Visualization, Validation, Methodology, Formal analysis, Conceptualization, Data curation. **Luca Kleineidam:** Writing – review & editing, Validation, Investigation, Data curation. **Melina Stark:** Writing – review & editing, Validation, Investigation, Data curation. **Oliver Peters:** Writing – review & editing, Resources, Investigation, Funding acquisition. **Julian Hellmann-Regen:** Writing – review & editing, Resources, Investigation, Funding acquisition. **Lukas Preis:** Writing – review & editing, Investigation. **Daria Gref:** Writing – review & editing, Investigation. **Josef Priller:** Writing – review & editing, Resources, Investigation, Funding acquisition. **Eike Jakob Spruth:** Writing – review & editing, Investigation. **Maria Gemenetzi:** Writing – review & editing, Investigation. **Anja Schneider:** Writing – review & editing, Resources, Investigation, Funding acquisition. **Klaus Fliessbach:** Writing – review & editing, Investigation, Conceptualization. **Jens Wiltfang:** Writing – review & editing, Resources, Investigation, Funding acquisition. **Claudia Bartels:** Writing – review & editing, Investigation. **Niels Hansen:** Writing – review & editing, Investigation. **Ayda Rostamzadeh:** Writing – review & editing, Investigation. **Emrah Düzel:** Writing – review & editing, Resources, Project administration, Investigation, Funding acquisition. **Wenzel Glanz:** Writing – review & editing, Investigation. **Enise Incesoy:** Writing – review & editing, Investigation. **Katharina Buerger:** Writing – review & editing, Resources, Investigation, Funding acquisition. **Daniel Janowitz:** Writing – review & editing, Investigation. **Sophia Stöcklein:** Writing – review & editing, Investigation. **Robert Perneczky:** Writing – review & editing, Resources, Investigation, Funding acquisition. **Boris-Stephan Rauchmann:** Writing – review & editing, Investigation. **Stefan J. Teipel:** Writing – review & editing, Resources, Investigation, Funding acquisition. **Ingo Kilimann:** Writing – review & editing, Investigation. **Christoph Laske:** Writing – review & editing, Resources, Investigation, Funding acquisition. **Sebastian Sodenkamp:** Writing – review & editing, Investigation. **Annika Spottke:** Writing – review & editing, Resources, Project administration, Investigation, Funding acquisition. **Marie Kronmüller:** Writing – review & editing, Project administration, Investigation. **Sandra Roeske:** Writing – review & editing, Investigation. **Frederic Brosseron:** Writing – review & editing, Investigation, Data curation. **Alfredo Ramirez:** Writing – review & editing, Investigation, Data curation. **Matthis Synofzik:** Writing – review & editing, Investigation, Data curation. **Matthias C. Schmid:** Writing – review & editing, Investigation, Data curation. **Frank Jessen:** Writing – review & editing, Resources, Project administration, Investigation, Funding acquisition. **Michael Wagner:** Writing – review & editing, Validation, Supervision, Investigation, Data curation, Resources, Funding acquisition.

## Declaration of competing interest

The authors declare the following financial interests/personal relationships which may be considered as potential competing interests:

Elizabeth Kuhn reports financial support was provided by Philippe Chatrier Foundation. Elizabeth Kuhn reports financial support was provided by 10.13039/501100023371Helmholtz Artificial Intelligence Cooperation Unit. Melina Stark reports financial support was provided by Hertie Network of Excellence in Clinical Neuroscience. If there are other authors, they declare that they have no known competing financial interests or personal relationships that could have appeared to influence the work reported in this paper.

## Appendix Supplementary materials

Image, application 1
